# Role of Cell Oxidant Status and Redox State in Controlling Cell Proliferation and Apoptosis in Two Models of Wallerian Degeneration of Rat Sciatic Nerve

**DOI:** 10.3390/antiox14101236

**Published:** 2025-10-15

**Authors:** Myrna Alexandra Roberta Dent, Alejandro Martínez-Gómez, Rolando Hernández-Muñoz

**Affiliations:** 1 Laboratorio de Neurociencias, Facultad de Medicina, Universidad Autónoma del Estado de México (UAMex), Paseo Tollocan y Jesús Carranza, Toluca 50180, Mexico; md@uaemex.mx (M.A.R.D.); amartinezgo@uaemex.mx (A.M.-G.); 2 Departamento de Biología Celular, Instituto de Fisiología Celular, Universidad Nacional Autónoma de México (UNAM), Mexico City 04510, Mexico

**Keywords:** crushed injury, transected injury, thymidine kinase, ornithine decarboxylase, NAD/NADH redox state, reactive oxygen species

## Abstract

After peripheral nerve lesion, the role of reactive oxygen species (ROS) has not been clarified during Wallerian degeneration. The present study examined the participation of oxidant stress after rat sciatic nerve injury induced by two experimental models (crush and transection). Here, biochemical parameters indicative of oxidant stress, nitric oxide (NO) metabolism, cell proliferation, apoptosis, and bioenergetics were determined in injured and contralateral sciatic nerves and caudofemoralis muscle. After crushing, we found two peaks of increased lipid peroxidation (LP) by-products and carbonylation of proteins in crushed nerves. In transected nerves, increases in LP showed similar patterns in both proximal and distal nerve. In both models, NO production was decreased and accompanied by an early increase in cell proliferation. Moreover, caspase-3 activity increased later only in crushed nerves. NAD availability and mitochondrial cytochrome oxidase activity were increased in transected but not in crushed nerves. The contralateral nerves also had changes in these parameters, but in a differential manner depending on the type of nerve lesion. In conclusion, present data suggest that changes in the patterns of LP may play a regulatory role in cell damage and death, somehow exerting a control in the progression of Wallerian degeneration.

## 1. Introduction

When a peripheral nerve is injured a series of events occur referred to as Wallerian degeneration, involving axonal degeneration, cytoskeletal collapse, and myelin sheath degradation that starts mechanically with fragmentation into small ovoid-like structures, resulting in unmyelinated repair Schwann cells [1,2]; this is followed by debris clearance by both Schwann cells and macrophages recruited from circulation [3,4]. Myelin clearance is critical for regeneration [5]. These repair cells proliferate form longitudinal cell strands, termed Bands of Büngner, that guide axon regrowth, ensuring remyelination of axons, reinnervation of their targets, and, ultimately, regeneration of the nerve [2,6,7]. All these metabolic and structural changes occur rapidly after nerve injury; however, the signaling pathways that initiate axon loss translating into actual dismantling of axons with orchestrated events between the intra-axonal and glial bodies remain as fascinating issues for investigation.

The role of reactive oxygen species (ROS) has not been clarified during Wallerian degeneration. For instance, after nerve crush, the number of myelinated fibers is significantly lower in vitamin E-deficient rat nerves, suggesting that the potent antioxidant vitamin E could be an important factor of the normal process of nerve regeneration [8]. In fact, early motor fiber degeneration of the rat sciatic nerve is accompanied by markedly diminished activity of the antioxidant enzymes superoxide dismutase and glutathione reductase and increased lipid peroxidation (LP), and nodal sprouting regeneration is more affected by deficiency of vitamin E [9]. Also, an increased level of LP is partly reduced by NO synthase (NOS) inhibition in the early ischemia–reperfusion of the sciatic nerve [10], which gives further support to the involvement of ROS and its by-products during Wallerian degeneration, as well as the participation of nitric oxide (NO).

NO is generated by three isoforms of NOS and appears to be a critical factor during degradation of myelin debris, allowing axon re-growth to the distal stump [11,12]. Neuronal NOS (nNOS) and the inducible NOS (iNOS) isoforms contribute to Wallerian degeneration and promote axonal regrowth, whereas the endothelial NOS (eNOS) isoform interferes with and inhibits axon regrowth [13]. This suggests that the release of NO might be important in supporting the needs of successful Wallerian degeneration and regeneration of peripheral nerves. However, high concentrations of NO results in damage of the myelin sheath and subsequent demyelination, playing an important role in inflammatory demyelinating diseases, and loss of myelin is the result of selective damage to axons, not to Schwann cells [11,14,15]. It has been proposed that NO is a switch that can maintain the homeostasis of oxidative stress in which a high level of NO promotes axon pruning while its reduction allows axons to regrow [16]. Therefore, both ROS and NO levels need to be regulated in the progression of axon degeneration/regeneration during Wallerian degeneration. In fact, LP occurring in living cells can also regulate several cellular processes, such as proliferation, differentiation, and apoptosis of normal and neoplastic cells [17].

Hence, we hypothesize that changes in the oxidant status and redox state are constituting triggers of signaling pathways that could be involved in the regulation of Wallerian degeneration of peripheral nerves. Therefore, the present work is concerned with long-lasting Wallerian degeneration, induced by crushing or transecting rat sciatic nerves, as well as its relationships with proliferative, apoptotic, and metabolic events taking place throughout the recovery period.

## 2. Material and Methods

### 2.1. Experimental Models

All procedures involving animals were carried out in accordance with the Official Mexican Norm for production, care, and use of laboratory animals (NOM-062-ZOO-1999). The protocol (48447-Q) was approved by the Bioethics Committee of the School of Medicine of the Universidad Autónoma del Estado de México, minimizing the number of animals used and their suffering.

Male Wistar rats (200–300 g, n = 120) were used for the experiments. The animals were grouped as follows: (1) twenty controls (intact nerves); (2) fifty crushed nerves (5 rats per each experimental point); (3) fifty transected nerves (5 rats per each experimental point); and (4) four sham-operated nerves (2 rats per experimental point).

Rats were anesthetized during surgery with ketamine (90 mg/Kg Chevinova, Mexico City, Mexico) and xylazine (10 mg/Kg Procin, Toluca, Mexico). All the surgical procedures were conducted under aseptic conditions [18]. Anesthesia was confirmed and monitored by the absence of a pain reflex. Surgery was performed followed by hair removal from the lateral face of the left hind paw. An incision, using a no. 23 scalpel blade (Hergom, Beijing, China), was made in the skin followed by divulsion of the gluteus maximus and separation of the biceps femoris at the mid-thigh level to uncover the sciatic nerve. For crush injury the nerve was crushed with fine forceps # 7 (Dumont, Montignez, Switzerland) for 30 s. To verify a complete sciatic nerve crush in rats, first we conducted a visual assessment, ensuring a clear gap and loss of continuity in the crush nerve, and postoperatively, we verify via checking for paralysis of the muscles in the toes. Rats exhibiting any toe movement are excluded as it indicates an incomplete crush. For transected injury, the sciatic nerve was cut, and the tip of distal stump was sutured with one stitch (nylon 4.0 Atramat, Mexico City, México) to the adductor magnis muscle to prevent proximal regenerating axons from entering the distal stump and to encourage nerve degeneration. Rats were allowed to recover for 1, 3, 5, 7, 10, 15, 20, 25, 28, and 32 days after crush and transection. Rats were killed by CO_2_ overexposure. Crushed, proximal, and distal stumps of transected nerves, contralateral nerves from both models, and normal rat sciatic nerves were excised. Normal sciatic nerves were used as control. We also did two points at 5 and 20 days of three nerves at each point of the sham-operated controls and found that there were no differences with normal rats. Therefore, we decided to use only normal rats as controls.

### 2.2. Obtaining Nerve Samples

The nerves were split into manageable strands and then gently homogenized in a 20 mM phosphate buffer solution (pH 7.4). The homogenates were spun at 1200 g for 15 min at 4 °C, and the supernatants were then used to determine parameters indicative of cell proliferation, apoptosis, and oxidant stress, as well as to quantify the activity of cytochrome oxidase. Another set of nerve strands were directly homogenized in cold-perchloric acid (8% *w*/*v*, final concentration) and centrifuged to remove denaturalized proteins. In the neutralized supernatants from these extracts, the redox-pair metabolites (lactate and pyruvate) were determined. The denaturalized nerve strands contained 5 to 7 mg of whole protein.

### 2.3. Sampling Other Organs

Homogenates (in phosphates buffer, pH 7.4) of brain, liver, and anterior (brachial) nerves, as well as contralateral and surrounding caudofemoralis muscles in the zone of the injured sciatic nerve, were also obtained at the time the injured sciatic nerves were excised and at all time points studied.

### 2.4. Assays for LP and Protein Carbonyl Groups

In samples from normal, injured, and contralateral nerves, the ROS by-product levels in sub-cellular fractions were estimated through the method described by Viarengo et al. [19]. The LP-related conjugated dienes were assessed as previously described [20], whereas the “production” of free radicals was determined by a technique based on luminol-chemiluminescence [21]. The protein carbonyl content in nerve preparations, as an index of oxidative damage, was estimated according to Levine et al. [22].

### 2.5. Estimation of DNA Synthesis and Compensatory Cell Proliferation During Wallerian Degeneration of the Sciatic Nerve

The cytosolic thymidine kinase (TK; EC 2.7.1.21) activity was determined by the technique of Sauer and Wilmanns [23], using [methyl-3H] thymidine (sp. act. 2 Ci/mmol; Dupont New England Nuclear Co., Boston, MA, USA). Ornithine decarboxylase (ODC; EC 4.1.1.17) activity was assayed by a radiometric technique using [1-^14^C]-ornithine (sp. act. 54 mCi/mmol) as the substrate [24].

### 2.6. Assessment of Apoptosis

In order to gain some insight into the onset of apoptosis, we measured the rate of apoptosis through determining the activity of caspase-3, assayed with a colorimetric kit (Millipore-Merck, Temecula, CA, USA), based on the hydrolysis of acetyl-Asp-Glu-Val-Asp p-nitroanilide (Ac-DEVD-pNA) by caspase-3, resulting in the release of the p-nitroaniline (pNA) moiety and contrasting the caspase activity using a specific caspase inhibitor (Ac DEVD-CHO) according the manufacture instructions. Caspase-3 activity is expressed as units of activity (1 unit corresponds to 1 nmol of cleaved substrate per hour).

### 2.7. Determination of Redox-Pair Cytosolic Metabolites, NO Metabolism, and Activity of Cytochrome Oxidase

Perchloric acid extracts obtained by freezing the clamped sciatic nerves were used for metabolite determinations by enzymatic methods for lactate and pyruvate [25]. In a homogenate of sciatic nerves, the activity of cytochrome oxidase (EC 1.9.3.1) was quantified by monitoring oxygen consumption [26]. The amount of NO produced was estimated by measuring the sum of nitrites and nitrates, as well as of citrulline, through colorimetric methods, as previously reported [27].

### 2.8. Calculations and Statistics

Cytosolic and mitochondrial redox states were calculated from the lactate/pyruvate ratio in accordance with Stubbs et al. [28]. Estimation of the NAD/NADH ratio from the cytoplasmic compartment was realized using the following equation: NAD^+^/NADH + H^+^ = [oxidized substrate]/[reduced substrate] • 1/*Keq* of lactate dehydrogenase (1.11 × 10^−4^ M). The data are reported as mean ± standard deviation (SD) for each group. All statistical analyses were performed using PRISM version 4.0 (GraphPad v. 10.4.1). Inter-group differences were analyzed by one-way ANOVA, followed by Tukey’s multiple comparison test as a post-test to compare the group means if the overall *p* < 0.01, and we considered this *p* as statistically significant.

## 3. Results

### 3.1. Production of ROS By-Products and Conjugated Dienes in the Injured Sciatic Nerve After Crushing or Transection

**Figure 1:**
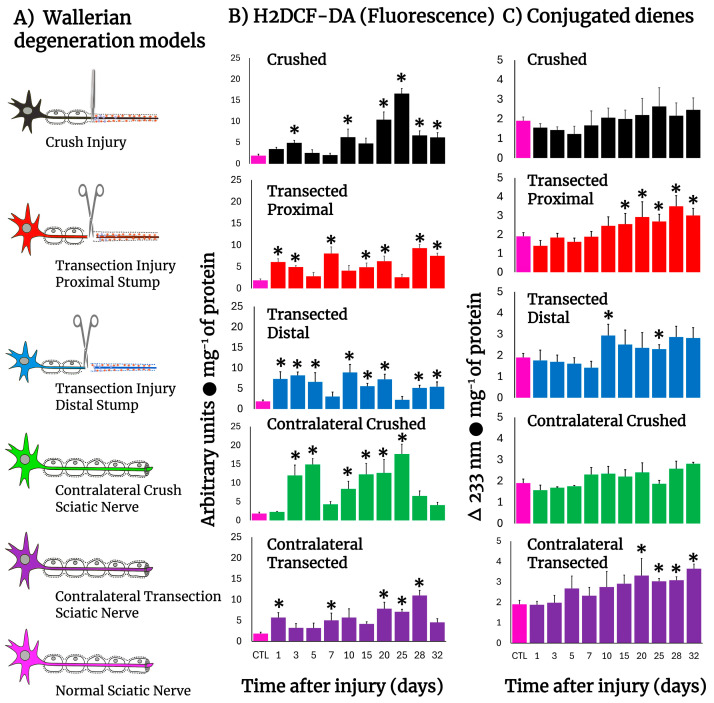
Production of ROS (H2DCF-DA fluorescence) and lipid peroxidation by-products in injured sciatic nerves after crush and transection and in the respective contralateral (intact) nerves. Results are expressed as mean ± SD of 5 determinations for each experimental point of the different models of nerve sciatic injury shown in panel (**A**) (controls: rose bars; crushed nerves: black bars; transected proximal nerves: red bars; transected distal nerves: blue bars; contralateral nerves (crushed): green bars, and contralateral nerves (transected): purple bars, for H2DCF-DA fluorescence products (panel (**B**)), and conjugated dienes (panel (**C**))). Statistical significance (*) *p* < 0.01.

Figure 1 shows schemes of the different experimental models of sciatic nerve injuries we used (Panel A). The crushed rat sciatic nerve increased the amount of LP, as assessed by the content of H2DCF-DA-reacting by-products, after injury (Figure 1B), eliciting mainly three peaks at 3, 10, and 25 days. The first two small peaks correspond to the time of repair cell proliferation and the time in which the bands of Büngner guide axon regrow, starting at day 20 and showing a maximum peak at day 25 (almost 10-fold over controls), which is the time when regeneration is taking place and axons make contact with their target (Figure 1B), suggesting a possible role of oxidative homeostasis activity during regeneration. After transection, both nerve sections rapidly increased the production of H2DCF-DA reacting by-products which normalized between 5 and 10 days. Both sections of the distal and proximal injured nerves increased LP again, maintaining LP up to day 20, with low oxidative activity throughout the nerve degeneration process, which is very different from the profile observed in crushed nerves. At later times (day 25), the crushed nerve shows an LP pattern that is a “mirror-image” of that obtained in the transected nerve (Figure 1B). Unexpectedly, contralateral (intact) nerves also showed enhanced levels of H2DCF-DA reacting by-products, which was more drastic in crushed nerves, showing a peak at day 5 and 25, similar to the ipsilateral crushed nerve, but of higher magnitude, suggesting that the oxidative stress is similar in both nerves, but it is not regulated as in the contralateral nerve; meanwhile, the profile was opposite regarding the time soon after injury, in which increased levels of LP followed the same patterns at later times in both transected and crushed nerves (Figure 1B).

Despite these observations, we also found increased amounts of conjugated dienes present in the cell membranes from injured nerves (Figure 1C); the patterns were different when compared to those of H2DCF-DA reacting by-products (Figure 1B). For instance, an enhanced rate of formed conjugated dienes was noted starting 10 days after injury and was maintained at high level in both models (Figure 1C). Unexpectedly, membrane-conjugated dienes were found to be significantly increased earlier in the contralateral nerves, mainly in those animals subjected to transection; in both cases, conjugated dienes did not return to control values (32 days after injury; Figure 1C).

### 3.2. H2DCF-DA Reacting By-Product Content in Other Tissues During Wallerian Degeneration of Crushed Nerves

Looking for whether other tissues were able to respond to the injured sciatic nerve, as a systemic response, we determined the rate of H2DCF-DA reacting by-products in the brain, liver, intact brachial nerve (right forelimb), and the caudofemoralis muscle surrounding the sciatic nerve from animals subjected to crush injury (Table 1). In the brain cortex non-significant changes were observed, while the brachial nerve showed a significant decrease in H2DCF-DA reacting by-product levels after crushing the homolateral sciatic nerve (Table 1). The liver only showed an unexpected increase in H2DCF-DA reacting by-products on day 7 after crushing, while the caudofemoralis muscle from the injured zone clearly had two peaks of enhanced H2DCF-DA reacting by-products (at 3 and 20 days after injury; Table 1). Hence, we also studied several parameters in the caudofemoralis muscles (from the injured and contralateral zones) obtained from rats subjected to crush injury (Figure 6).

### 3.3. Production of Free Radicals Detected by Chemiluminescence in Crushed or Transected Sciatic Nerves

**Figure 2:**
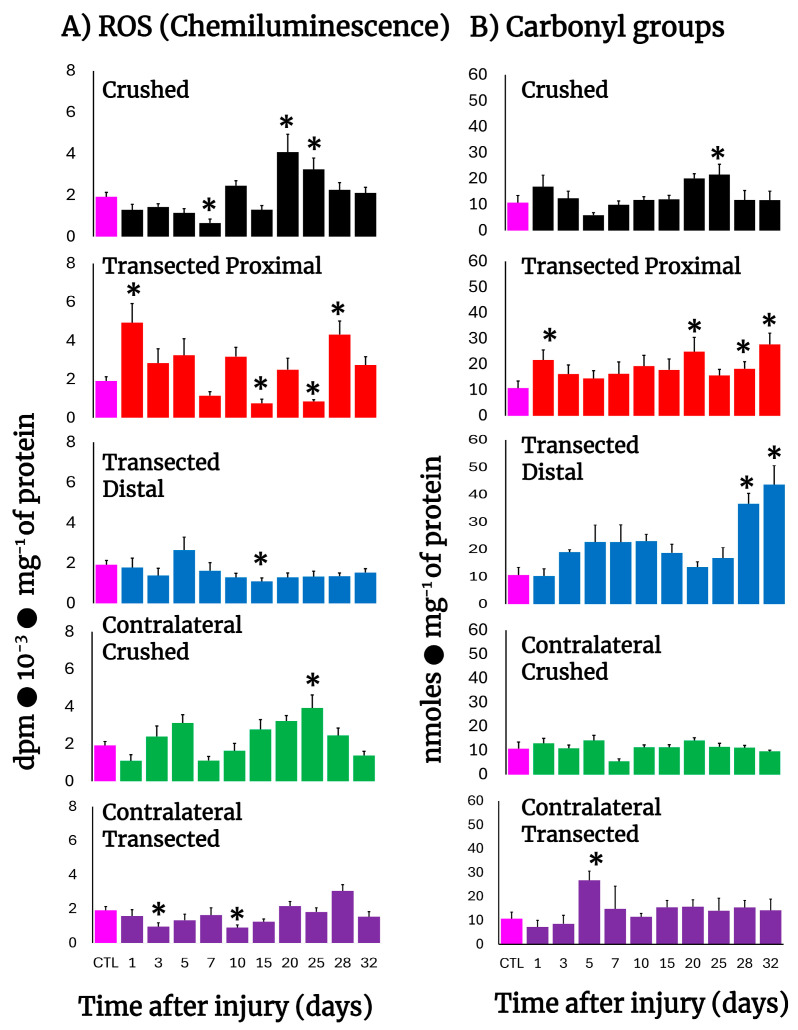
Production of ROS (chemiluminescence) and protein carbonyl groups in injured sciatic nerves after crush and transection and in the respective contralateral (intact) nerves. Results are expressed as mean ± SD of 5 determinations for each experimental point of the different models of nerve sciatic injury shown in Figure 1 and defined by the colored bars, for ROS-produced chemiluminiscence (panel (**A**)) and for carbonyl groups in proteins (panel (**B**)). Statistical significance (*) *p* < 0.01.

After transection, production of free radicals barely increased in proximal and distal nerves, except for the first day post-injury in the proximal nerves (Figure 2A), clearly contrasting with the higher rates of H2DCF-DA reacting by-products found in these nerves (Figure 1B,C), while crushed nerves show a significant peak in free radicals’ production on day 20 (Figure 2A). Interestingly, the contralateral nerves from crushed nerves have two peaks of chemiluminescence at 5 and 25 days after injury (Figure 2A), a phenomenon not present in transected nerves that show a lower production of free radicals even when compared with the control group (Figure 2A).

### 3.4. Rate of Protein Oxidation (Carbonyl Groups) in Crushed or Transected Nerves

Very early after transection both the proximal and distal nerves presented an enhanced content of carbonyl groups in proteins, indicative of their oxidation. Both profiles (proximal vs. distal) were very similar, except on day 5, where a mirror-image was observed (Figure 2B). In contrast, crushed nerves show a similar rate of oxidized proteins to control nerves (up to day 10), but a small peak of carbonyl protein groups was recorded on day 25 and declined thereafter (Figure 2B). As could be anticipated, in the contralateral nerves we did not find enhanced oxidation of sciatic nerve proteins, except for an unexpected sharp peak noted on day 5 after transection (Figure 2B).

### 3.5. NO Metabolism in Crushed and Transected Sciatic Nerves

**Figure 3:**
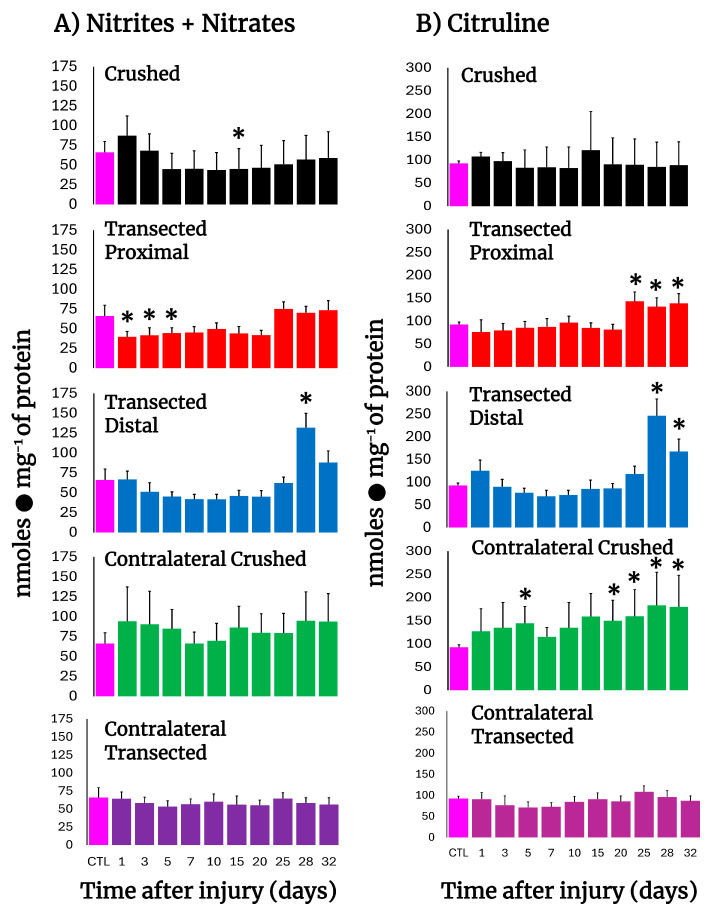
Production of NO by-products in injured sciatic nerves after crush and transection and in the respective contralateral (intact) nerves. Results are expressed as mean ± SD of 5 determinations for each experimental point of the different models of nerve sciatic injury shown in Figure 1 and defined by the colored bars for nitrates + nitrites sum (panel (**A**)), as well as for citrulline (panel (**B**)). Statistical significance (*) *p* < 0.01.

To assess NO production in injured nerves, the levels of nitrates, nitrites, and citrulline were determined (Figure 3). In both crushed and transected nerves, a consistent decrease in nitrates + nitrites was observed, suggesting diminished NO production, which slowly normalized at the end of the experiment and had a peak at day 28 in the distal stump (Figure 3A). Interestingly, in the other major product from NO synthase, namely citrulline, normal levels were found in the injured nerves, and a peak was also observed on day 28 in the distal stump (Figure 3B). As for the contralateral nerves, the sum of nitrites and nitrates was non-significant, different from the control samples, in both models of injury (Figure 3B). For citrulline, the levels were maintained higher when compared to the control in crushed nerves, while in transected nerves the citrulline levels were practically unaltered (Figure 3B). In conclusion, the results show a decrease in NO metabolism in injured nerves.

### 3.6. Parameters Indicative of Cell Proliferation in Crushed and Transected Sciatic Nerves

**Figure 4:**
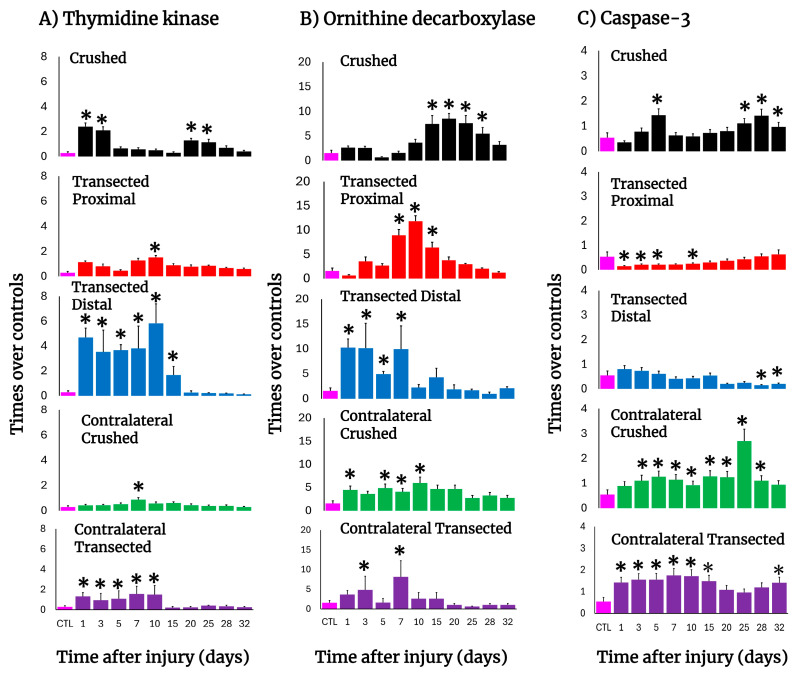
Parameters are indicative of cell proliferation and apoptosis in injured sciatic nerves after crush and transection and in the respective contralateral (intact) nerves. Results are expressed as mean ± SD of 5 determinations for each experimental point of the different models of nerve sciatic injury shown in Figure 1 and defined by the colored bars for the thymidine kinase (TK; panel (**A**)) and ornithine decarboxylase (ODC; panel (**B**)) activities, as well as for active caspase-3 (panel (**C**)). The control values were TK: 0.29 ± 0.11 nmols of formed [^3^H]TMP • hour^−1^ • mg^−1^ of protein, ODC: 1.56 ± 0.58 nmols • min^−1^ • mg^−1^ of protein, and caspase-3: 0.54 ± 0.19 units (1 unit corresponds to 1 nmol of cleaved substrate per hour). Statistical significance (*) *p* < 0.01.

In crushed nerves, there was an early elevation in the TK activity, reflecting active DNA synthesis, which suddenly decreased on day 5 but showed a second peak on day 20 and progressively returned to the control level (Figure 4A). After transection, a drastic increase in TK activity was also observed on day 1 in both the distal and proximal stumps but was more dramatic in the distal nerve (more than 12 times; Figure 4A). In fact, DNA synthesis in the distal nerve remained enhanced during the first 10 days after transection, declining on day 15; on the contrary, the proximal nerve showed a second peak of TK activity (day 10) which returned very slowly to the control levels for TK activity (Figure 4A). Unexpectedly, the contralateral nerves also changed TK activity. After crushing, a small peak on day 7 was observed (Figure 4A); in contrast, early after transection the TK activity drastically increased in the contralateral nerve, remaining high and decreasing on day 15 (Figure 4A).

The activity of ODC (polyamine synthesis), was also increased after nerve injury, but it had a different pattern compared to TK activity along the times tested during Wallerian degeneration (Figure 4B). In crushed nerves, ODC activity increased starting from day 10 and had a peak by day 20 returning to normal levels by day 30. As for transection, the distal nerve depicted an early increase in ODC activity, followed by two more peaks on days 7 and 15, then returning to the control range (Figure 4B). The proximal nerve had two peaks of maximum ODC activity on days 3 and 10 and normalizing by day 25 after surgery (Figure 4B). Again, contralateral nerves showed significantly enhanced ODC activity up to day 25 after crushing, whereas after transection, only two significant peaks on days 3 and 7 were observed (Figure 4B).

### 3.7. Activity of Caspase-3 in the Injured Sciatic Nerve After Crushing or Transection

The activity of caspase-3, as a reliable marker for apoptosis, was measured in both models (Figure 4C). Early after crushing, the injured nerve had significantly increased caspase-3 activity on day 5, returning to the control range and then progressively increasing with a peak of maximum activity on day 28, remaining significantly increased thereafter (Figure 4C).

In contrast, in both the proximal and distal transected nerves the level of caspase-3 activity was low or significantly lower than the controls (Figure 4C). Unexpectedly, in contralateral nerves the activity of caspase-3 was increased in both experimental models (2.6 to 3.0 times over control nerves, *p* < 0.01, during the 10 first days; Figure 4C).

### 3.8. Changes in the Cell Redox State (Cytoplasmic) in the Injured Sciatic Nerve After Crushing or Transection

**Figure 5:**
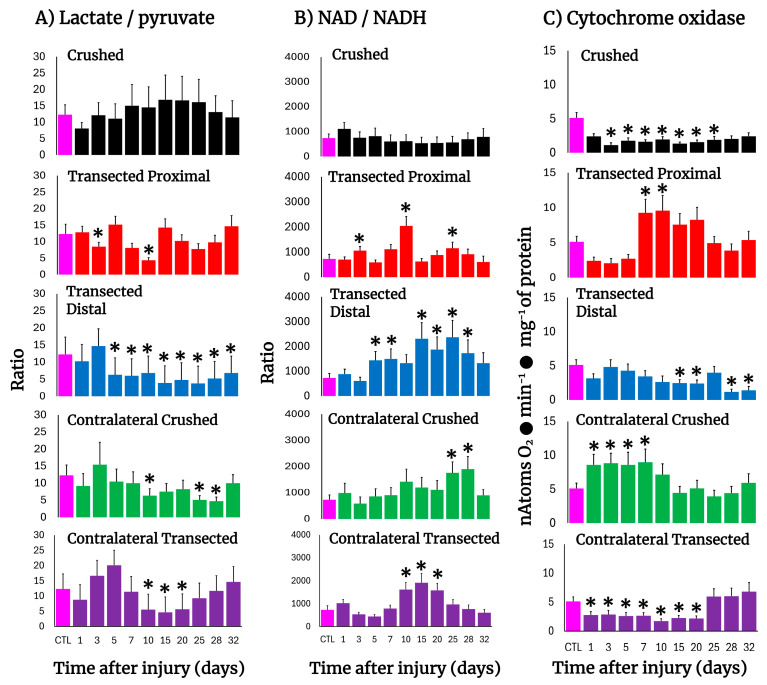
The lactate/pyruvate ratio, cytoplasmic NAD/NADH potential, and cytochrome oxidase activity in injured sciatic nerves after crush and transection and in the respective contralateral (intact) nerves. Results are expressed as mean ± SD of 5 determinations for each experimental point of the different models of nerve sciatic injury shown in Figure 1 and defined by the colored bars for the lactate/pyruvate ratio (panel (**A**)) and the NAD/NADH ratio (panel (**B**)), as well as for the activity of cytochrome oxidase (panel (**C**)). Statistical significance (*) *p* < 0.01.

The Wallerian degeneration achieved by crushing or transecting the sciatic nerve was accompanied by changes in the tissue concentrations of lactate and pyruvate, hence modifying the cytoplasmic redox NAD/NADH potential (Figure 5). After crushing, the injured nerve had a lower lactate/pyruvate ratio which was reflected in an increased NAD/NADH ratio (day 1); however, this more oxidized cytoplasmic redox state was rapidly normalized thereafter (Figure 5A,B).

On the contrary, after transection both the proximal and distal nerves showed lower lactate/pyruvate ratios, which led to a much-oxidized cytoplasmic redox state along the experimental times tested, which was strongly the case in the distal nerve (Figure 5A,B). In contralateral nerves, the NAD/NADH ratio was largely increased in crushed and transected nerves, representing a more oxidized cytoplasmic redox state, (Figure 5A,B), which was preceded by a discrete reduction of this redox state and then returned to control values (Figure 5A,B).

### 3.9. Mitochondrial Cytochrome Oxidase Activity in the Injured Sciatic Nerve After Crushing or Transection

The activity of cytochrome oxidase can partially reflect mitochondrial metabolism, and this enzyme activity has been used to assess neurogenesis [26]. Crushed nerves showed a drastic reduction in cytochrome oxidase activity which remained low up to the end of the experiment (Figure 5C). A similar pattern was observed in the distal nerve after transection, while the proximal nerve showed a significant drop in the cytochrome oxidase activity until day 5, followed by enhanced levels of activity of this enzyme (Figure 5C). As to the contralateral nerves, crushed nerves showed an increase in mitochondrial activity until day 10, normalizing thereafter (Figure 5C), while in transected nerves, an unexpected decrease in this mitochondrial enzyme activity was recorded up to day 20 after surgery, returning later to control values (Figure 5C).

### 3.10. Parameters Are Indicative of Oxidant Stress, Proliferation, and Apoptosis in Leg Muscles After Crushing the Right Sciatic Nerve

**Figure 6:**
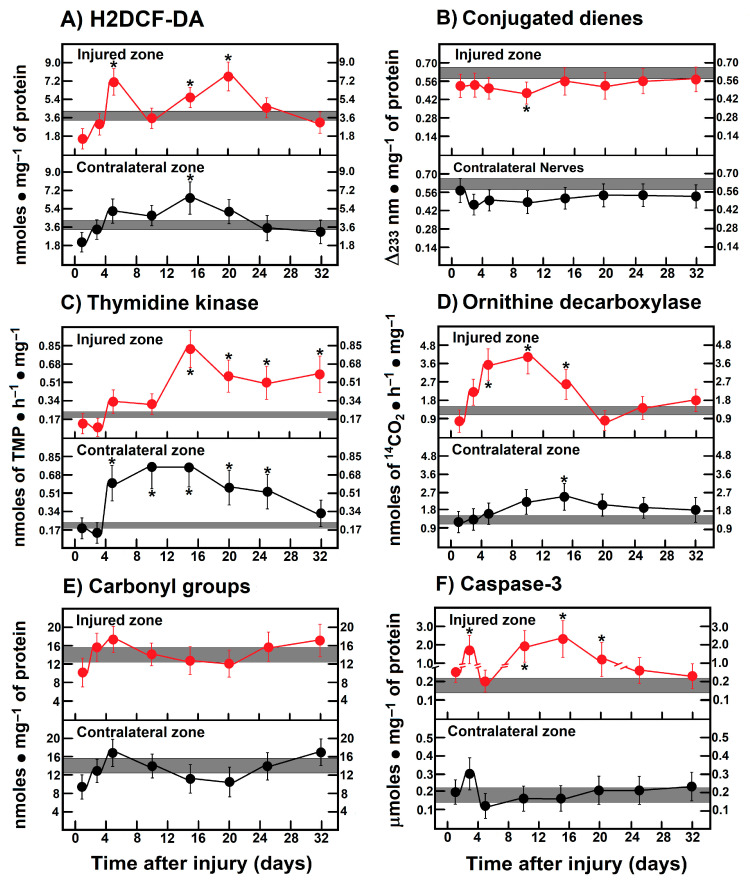
Parameters indicative of oxidant stress, proliferation, and apoptosis in caudofemoralis muscle after crushing the right sciatic nerve. The results are expressed as mean ± SEM of 5 determinations for each experimental point. Number of dienes correspond to those conjugated in membrane layers, and carbonyl(s) groups correspond to oxidized proteins. The shadowed horizontal bar is the control range, and symbols in red represent muscle homogenates from the injured zone, as well as those from the contralateral zone (black circles). H2DCF-DA: 2′,7′-dichlorodihydrofluorescein diacetate. Statistical significance (*) *p* < 0.01.

As mentioned before, the caudofemoralis muscle clearly had two peaks of enhanced H2DCF-DA reacting by-products on day 5 and 20 after injury, which did not correlate with either conjugated dienes or carbonyl protein groups that did not change (Figure 6A,B,E). On the contrary, the contralateral muscle showed a low rate of oxidant changes compared to control nerves, except for the increased H2DCF-DA reacting by-product levels found on day 15 (Figure 6A). However, the activity of TK and ODC in the muscles from both the crushed and the contralateral zone showed a progressive increase (Figure 6C,D), but its time-course was quite opposite (mirror-image) to that found in injured nerves (Figure 6C,D). In the contralateral muscle, TK activity was also increased even at earlier times after crushing; on the contrary, ODC activity was slightly increased on day 10 (Figure 6C,D). Interestingly, activity of caspase-3 was robustly increased in the muscle from the injured zone showing two peaks at days 3 and 15, while in the contralateral zone, caspase-3 activity was not found significantly modified (Figure 6F).

### 3.11. Correlations Among Parameters Indicative of Oxidant Stress, Cell Proliferation, Apoptosis, and Onset of Mitochondrial Biogenesis in Crushed and Transected Sciatic Nerves with Their Respective Contralateral Nerves

**Figure 7:**
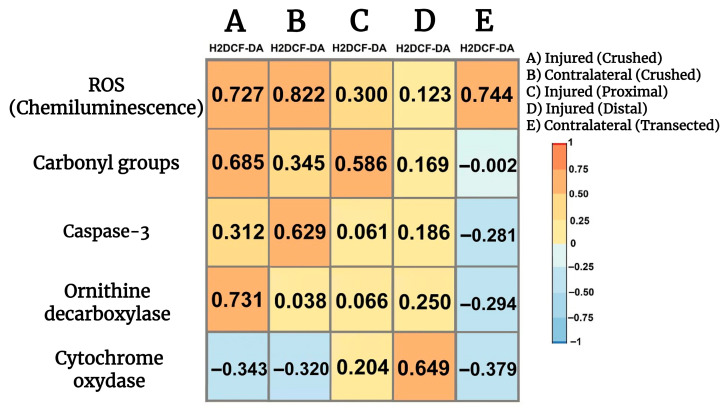
Correlations among parameters indicative of oxidant stress, cell proliferation, apoptosis, and onset of mitochondrial biogenesis in injured sciatic nerves after crush and transection and in the respective contralateral (intact) nerves. Heat maps show the relation between two sets of data. This relation is summarized with the Pearson’s correlation coefficient (r) for each relation in each scatter plot: H2DCF-DA fluorescence vs. ROS (chemiluminescence), against carbonyl groups, vs. caspase-3 activity, against ODC activity, and finally H2DCF-DA fluorescence vs. cytochrome oxidase activity.

We looked for possible relationships between parameters indicative of oxidant stress, namely H2DCF-DA reacting products, and cell proliferation, apoptosis, and mitochondrial function. Fluctuations in the production of LP by-products highly correlated with ROS generation in both crushed and contralateral nerves (Figure 7), while a lower direct correlation was noted in the transected proximal injured nerve (Figure 7) and a significant correlation was not found when examining the transected distal injured nerve (Figure 7). Production of H2DCF-DA reacting compounds also correlated well with the presence of carbonyl groups in proteins (oxidized) and, to a lower extent, the corresponding contralateral nerve, and a similar correlation was found in the transected proximal nerve (Figure 7). On the contrary, no significant correlations were observed in the transected distal nerve nor in the corresponding contralateral nerves (Figure 7). The H2DCF-DA fluorescence also correlated with active caspase-3 in the crushed nerve, and surprisingly, in the contralateral nerve there was an even better correlation between LP by-products and caspase-3 activity (Figure 7). We did not find a significant correlation between these two parameters in the transected proximal nerve or the distal nerve (Figure 7); interestingly, the contralateral nerves showed a weak but significant correlation between H2DCF-DA fluorescence and caspase-3 activity (Figure 7). As regards cell proliferation (most probably Schwann cells and macrophages), the H2DCF-DA fluorescence correlated with ODC activity in the crushed nerve, whereas, as might be anticipated, no correlation was recorded in the contralateral nerve vs. ODC. Meanwhile, in the transected proximal nerve we did not note any correlation between these parameters (Figure 7), in the transected distal nerve a weak but significant straight correlation was found between H2DCF-DA fluorescence and ODC activity. Again, the contralateral nerves of the transected injured nerves had a weak but inverse relationship between these parameters (Figure 7); in the transected distal nerve a weak but significant straight correlation was found between H2DCF-DA fluorescence and ODC activity. Again, the contralateral nerves of the transected injured nerves had a weak but inverse relationship between these parameters (Figure 7). Increased presence of LP by-products inversely correlated with the cytochrome oxidase activity; this effect is also observed in the corresponding contralateral nerve (Figure 7). On the contrary, we found a weak direct correlation between H2DCF-DA fluorescence and cytochrome oxidase in the transected proximal nerve, while the injured distal nerve had a robust inverse correlation between these parameters (Figure 7). Interestingly, the corresponding contralateral nerves showed a significant inverse correlation between H2DCF-DA fluorescence by-products and the cytochrome oxidase activity (Figure 7).

## 4. Discussion

After nerve crush injury or axonotmesis, the nerve structure remains intact, allowing regeneration over time, while transected nerves or neurotmesis completely severs the nerve. The former is a regulated process in which, firstly, the distal stump of the nerve undergoes Wallerian degeneration to remove axons and myelin fragments and, secondly, a permissive environment is generated for nerve regeneration. In contrast, transected nerves undergo complete Wallerian degeneration without being able to regenerate [29,30].

The involvement of free radicals and LP during the metabolic adjustment occurring in proliferating tissues is an important issue. It has been established that the close relationship between the periodicities of thymidine kinase activity and decreased microsomal LP has some role in modulating the cell division process (Figure 1 and Figure 3) [31]. This agrees with the low NAD phosphate-dependent microsomal LP exhibited in tissues that have a substantial rate of cell division (Figure 1 and Figure 3) [32]. It is known that ROS is involved in the toxic effects produced by various agents on many cellular systems, but it is also accepted that low levels (“low tone”) of ROS could control factors involved in cell homeostasis [33].

Since one of the limitations is using a non-specific H_2_DCF-DA assay for ROS detection, we performed four assays indicative of oxidative stress as well as oxidative changes in lipids and proteins (Figure 1 and Figure 2). Moreover, the detection of ROS by chemiluminescence (Figure 2) is based on the reaction of the “O” form of xanthine oxidase, which confers more specificity to superoxide radicals. The results from these parameters are indicative of oxidant activity and clearly show changes in ROS by-products in crushed nerves, while transected nerves remained lower during Wallerian degeneration (Figure 1).

Regarding LP by-products and protein oxidation (Figure 1), an early increase was observed in samples from crushed or transected nerves. However, the secondary increases found in ROS by-products did not correlate so well with the rate and pattern of protein oxidation but rather were more related to parameters indicative of active cell proliferation (Figure 1 and Figure 3). This phenomenon has been previously observed by our research group in tissues with high rates of cell proliferation such as liver and gastric mucosa, where LP was quantitatively distinct among sub-cellular fractions in both experimental models [20,34].

During peripheral nerve regeneration, quiescent mitotic Schwann cells proliferate quickly in the distal nerve and convert to repair Schwann cells to form the regeneration tracks or Büngner’s bands, which provide the pathway to direct axons to their targets [1]. Our data clearly demonstrated that oxidant stress (ROS by-products) could also regulate proliferation of Schwann cells, with temporality and in magnitude correlated with ODC activity (Figure 1, Figure 3 and Figure 6). This might indicate that active synthesis of polyamines is a step involved in axonal regeneration during Wallerian degeneration [35]. A polyamine-stress response, as in the mature brain, appears to be a constructive reaction implicated in neuronal cell death and hypoxia-induced brain cell damage [36]. ODC expression and activity can be controlled through ROS [37], as well by an increased calcium efflux accompanied by decreased PKC activity [38]. Also, muscle hypertrophy and muscle fiber regeneration have been associated with an initial increase in polyamine levels through enhanced ODC activity [39]. In this context, the ODC activity in the ipsilateral muscle of the injured nerve also seemed to be triggered by increased LP by-products (Figure 5).

In experimental models with well-defined temporal patterns of cell cycle progression, LP has emerged as a potential regulator of proliferative activity. Within the context of post-hepatectomy liver regeneration, LP may act as a modulatory factor, influencing both the initiation and cessation of mitosis in the regenerating liver, thereby modifying the magnitude and timing of the proliferative response [34]. Furthermore, oxidative stress and free radical-mediated processes might trigger a generalized cellular response, activating transcription factors that function as signal transducers between the cytoplasm and nucleus. This framework supports a critical role for LP during the early stages of liver proliferation. For example, the presence of LP-related products in the cytosol could modulate the activity of ornithine decarboxylase (ODC), a key enzyme in polyamine biosynthesis, which is essential for effective liver regeneration [36,40], which is linked to an enhanced activation of STAT proteins, mainly as activated STAT-3, significantly changing the cytoplasmic pool for STATs [41]. Therefore, the latter could be considered as an exemplary mechanism of ROS/LP in the present experimental models.

During Wallerian degeneration Schwann cells clear myelin debris through phagocytosis and by recruiting macrophages to the site of injury, a process dependent on the breakdown of the blood–nerve barrier. Macrophages themselves produce factors to promote Schwann cell proliferation [4,42]. A large body of evidence points to macrophages, present in high numbers in the inflamed peripheral nerve, and Schwann cells as the predominant source of NO, through the expression of iNOS [43,44]. The release of NO following peripheral nerve injury seems to be a crucial factor in the successful degeneration/regeneration process, and enhanced expression of iNOS is involved in the clearance of axon and myelin breakdown prior to regeneration [45]. However, excessive local levels of NO during inflammation may damage axons and growth cones [46]. High levels of iNOS-mediated production of NO may be involved in the production of detrimental effects in the recovery, favoring PNS cell-mediated demyelination [47] and higher concentrations of NO, which can cause strand breaks and fragmentation in the DNA of target cells [48]. This suggests that a balance between NO production and axon regrowth needs to be maintained and is necessary to neutralize local NO to save axons from NO mediated degeneration and to support axon growth. This is also because not all axons and Schwann cells are at the same stage of degeneration/regeneration, because Wallerian degeneration is an asynchronous process [49].

Our results indeed showed that Wallerian degeneration is accompanied by changes in NO metabolism, which was unexpected. During the first day, nitrates, nitrites, and citrulline (to a lesser magnitude) were slightly increased in crushed nerves, and this amino acid was also increased in the distal stump early after transection (Figure 2). However, in the injured nerves, nitrites and nitrates were strongly decreased over the time studied which could suggest that progression of Wallerian degeneration requires low levels of NO production.

It is well known that in response to nerve injury, axons and myelin are degraded by the cooperative action of Schwann cells and macrophages, followed by Schwann cell division, with the highest rate of multiplication reached by day 3 to 7 after lesion and decreases after that [50,51]. This process is also enhanced by the increase in ornithine decarboxylase (ODC) activity [12,52]. Our results with thymidine kinase show a progressive increase in proliferation in the distal stump with a 6-times increase on day 10, and in the proximal stump a small increase only on day 10 is observed. The results on the transected nerves correlate with the high increase in ODC activity on both stumps.

However, in crushed nerves there is a small increase in TD on days 1 and 3, but then it decreases below the control values and rises again on day 20 and 25. This result does not agree with what has been reported [49] and with what we have observed in our laboratory, where an increase initiates on day 1 and increases massively on day 3 to day 7 followed by a decreasing frequency for 2.3 weeks. Purines are synthesized via two principal routes: the de novo and salvage pathways [53,54]. Thymidine kinase is involved in the salvage pathway of DNA synthesis and correlates with the proliferative activity of transected nerves (Figure 3A). However, a possible explanation for the lack of TK activity in crushed nerves might be that proliferation is carried out by a de novo pathway. The main limitation of this pathway is that it is a slower process and metabolically very costly, but it is tightly regulated through multiple mechanisms [55]. In a crush nerve, both degeneration and regeneration processes take place; therefore, the process must be highly regulated to be carried out, and the de novo pathway may be suitable as it might allow regulation of the regeneration process.

It is known that Schwann cells prepared from degenerating axons show differentiation and finally undergo spontaneous apoptosis in vitro [56]. Nonetheless, neurons appear to have at least two self-destruct programs, presenting the “classical” caspase-dependent apoptotic program, with the participation of caspase-3 as the final step responsible for apoptotic neuronal death during Wallerian degeneration, and another for selective axon degeneration [57,58]. Our data could support that statement, since the neuronal cell death seemed to depend on the milieu or microenvironment where Wallerian degeneration takes place, and support the view that caspases do not contribute to Wallerian degeneration. Caspase activation is not detected during axonal degeneration, although it is activated in a neuronal dying cell, and caspase inhibitors do not block or retard axon degeneration but inhibit apoptosis of neurons [57].

For instance, in crushed nerves, it was clear that caspase-3 activity progressively increased with a peak on day 25 (Figure 4C). It is possible that at the beginning when degeneration is taking place, only a small number of cells died by apoptosis, but later, when degeneration is over, the excess of Schwann cells or macrophages died by apoptosis (Figure 3). Oppositely, in both proximal and distal stumps of the transected sciatic nerve, despite that Schwann cell death could occur, the caspase-3 activity was not significantly different from that of controls (Figure 4C). These results suggest that there is more than one mechanism for controlling the number of dedifferentiated Schwann cells. Surprisingly, and independent of the lesion type, contralateral nerves depicted an early and sustained activation of the pro-apoptotic caspase-3 (Figure 4C), which is very different from the injured nerve, suggesting that while in the injured nerve the process is highly regulated, it is different in the contralateral nerve. It would certainly be nice to follow up this study with supporting evidence from additional apoptosis markers.

In fact, there is increasing evidence that unilateral nerve lesions affect both injured (ipsilateral) and uninjured (contralateral) nerves. Although, the neuroanatomy does not show connections between neurons that innervate homologous areas of the right and left sides of the body. These effects are qualitatively like those occurring at the ipsilateral side, but of small magnitude and time course [58,59]. The biological significance of these contralateral effects is unclear, but the existence of these changes implies the presence of unrecognized signaling mechanisms that link the two sides of the body [59]. This bilateral communication also shows that the effects are not systemic. In this study, most of the parameters we studied show changes in the contralateral nerves. It has been suggested that in different models of nerve injury invasion of macrophages into the contralateral dorsal root ganglion may be mediated by lost motor neurons or by interneurons, responding to retrograde transport of factors produced during Wallerian degeneration or their delivery by blood flow [60]. In transected rat nerves, a profound, long lasting, nerve-branch-specific loss of distal innervation is present in both contralateral and ipsilateral nerves, even after 5 months [60]. Also, some patients after unilateral injury develop contralateral changes including limb edema, loss of strength, and changes in bone metabolism [61]. In the degeneration of retina ganglion cells, the contralateral uninjured retinas show molecular changes, significant neuronal death, and glial activation, suggesting that the bilateral communication might be orchestrated by the spinal cord [62]. Therefore, despite it arguing against a peripheral mechanism (blood-borne circulating factors), it is more likely to be, although it needs to be proven, a central mechanism, probably signaling via the system of commissural interneurons that is present in spinal cord and brainstem.

It was very surprising to find that contralateral nerves had a drastic increase in parameters indicative of oxidant stress and cell proliferation, which closely followed the time course of those seen in injured nerves. However, opposite patterns were also noted, mainly regarding NO production and apoptotic events, between injured and contralateral nerves. We think that this is the first demonstration that changes underlying progression of Wallerian degeneration are closely reflected in the contralateral side, in a long follow-up study. Hence, despite considering that excessive NO formation accelerates LP, as well as axonal degeneration in the sciatic nerve [10], changes in LP and antioxidant status are related with nerve fiber degradation in the neuropathy induced by some toxins [63], or in diabetic neuropathy [64], and we believe that LP is also serving as a signaling mechanism for Wallerian degeneration. Our statement is supportive of changes occurring in the contralateral nerve without evidence of damage, as well as the findings in the ipsilateral and contralateral leg muscles surrounding the intact and injured sciatic nerves.

It has been suggested that Wallerian degeneration is regulated by NAD-dependent processes and might exert its effects through the SIRT1 pathway [65], and genetic studies have attributed a slow Wallerian degeneration phenotype (Wlds) to the overexpression of Nmnat1 [66]. These findings suggest the importance of a NAD-dependent process in axon protection. Our results show important changes in NAD availability and on cytoplasmic redox potential during the progression of crush or transection-induced Wallerian degeneration (Figure 4B). In the distal stump of the transected sciatic nerve, NAD availability was increased along the time studied (Figure 4B), as reflected by a significant lower lactate/pyruvate ratio, whereas the proximal portion of the injured nerve essentially had two maximum peaks of increased NAD availability at 10 and 25 days after transection, respectively (Figure 4). The fact that, in the model of crush-induced sciatic nerve injury, the NAD/NADH ratio was not increased during progression of Wallerian degeneration would suggest that, besides the already commented effect of NAD in protecting nerve viability, this parameter (cytoplasmic NAD/NADH ratio) might be consistent with possible changes in the relative contribution of metabolic pathways providing cellular energy.

In addition, deprivation of ATP blocks the axon retraction caused by inhibition of microtubule assembly, which suggests that axon retraction is an active process that requires intimate interaction between actin and the microtubule cytoskeleton [67]. Moreover, the notion that distal portions of the transected axons die passively for lack of nutritional support has been challenged by the fact that distal axons can survive for a long time after transection in the Wlds mice [68]. In this context, our data revealed that during Wallerian degeneration, mitochondrial metabolism was readily diminished, as apparently reflected by decreased activity of cytochrome oxidase. The latter, associated with an increased cytoplasmic NAD/NADH ratio, could lead us to suggest that support of metabolic energy, in the form of ATP availability, is mainly accounted for by a very active glycolytic pathway. However, it can also be a signal for increasing mitochondrial oxidative metabolism at the onset of Wallerian degeneration (Figure 4C). It is known that there is no re-innervation of muscles, or subsequent maturation of the regenerating motor nerve fibers during progression of Wallerian degeneration [69], and myotonia is diminished and disappears in muscles shortly after the nerves have undergone Wallerian degeneration [70]. In this study, it was clear that the surrounding caudofemoralis muscle isolated from both the injured and contralateral nerves presented striking responses to that which is happening in the sciatic nerves, even when it is not the nerve target for re-innervation. This tissue showed an increase in LP, dramatically accompanied by enhanced activities of enzymes indicative of cell proliferation and apoptosis. On the other hand, in the contralateral muscle, TK and ODC activities were significantly increased, despite that an augmented antioxidant capacity was seen in this tissue. The occurrence of these changes in intact non-proliferating tissue strongly suggests that molecular signals acting in the nerves flow to the surrounding muscle and respond to the damage. The results also show a different response pattern of injury between transected and crushed nerves during Wallerian degeneration/regeneration. Differences between these two types of injury have already been shown; Wallerian degeneration is a rapid, asynchronous, progressive, and wave-like process, but it can change its orientation depending on the lesion type [71,72]. All these differences correlate well with the differential changes in oxidative, proliferative, and metabolic events reported here.

Our study has several limitations. Although this study explored several cellular processes that may be involved in Wallerian degeneration, further investigation of these possible events is required, especially to clearly explain what occurs in the contralateral nerves.

## 5. Conclusions

In conclusion, (1) ROS/Lipid peroxidation may regulate Wallerian degeneration, (2) crush vs. transection lesions show distinct metabolic and apoptotic profiles, and (3) contralateral effects are novel and interesting, which warrant further study. The data presented here also strengthen our hypothesis that changes in the patterns of LP may play a regulatory role in in the progression of Wallerian degeneration and are not merely a consequence of cell damage and subsequent death. Further research is needed to demonstrate this.

## Figures and Tables

**Table 1:** Rate of lipid peroxidation in several organs after crushing the right sciatic nerve.

Parameter	Lipid Peroxidation (Arbitrary Units *•* 10^2^ *•* mg^−1^ of Protein)			
Organ	Brain Cortex	Brachial Nerve	Liver	Muscle
Controls	5.8 ± 1.6	2.7 ± 0.7	1.3 ± 0.4	1.4 ± 0.5
**Time after Surgery**				
Day 1	6.1 ± 1.8	2.3 ± 0.7	1.2 ± 0.4	3.0 ± 0.7 *
Day 3	6.2 ± 2.0	1.7 ± 0.4 *	1.3 ± 0.4	4.1 ± 0.4 *
Day 5	6.1 ± 4.0	1.3 ± 0.4 *	1.2 ± 0.2	1.4 ± 0.4
Day 7	6.0 ± 4.0	2.0 ± 0.7	3.3 ± 1.3 *	1.6 ± 0.4
Day 10	5.9 ± 4.0	2.7 ± 1.1	2.0 ± 0.7	1.7 ± 0.5
Day 15	6.0 ± 1.8	1.8 ± 0.7	1.5 ± 0.4	2.4 ± 0.7 *
Day 20	5.7 ± 1.8	1.6 ± 0.7	1.4 ± 0.4	3.0 ± 0.7 *

The results are expressed as means ± SD of 5 individual observations per experimental point for lipid peroxidation (LP) by-products identified by the fluorescence generated by the complex with the 2′,7′-dichlorodihydrofluorescein di-acetate (H2DCF-DA) probe. The braquial nerve corresponded to the main nerve found in the anterior rat’s paw (“arms”). Statistical significance (*) *p* < 0.01 against the control values.

## Data Availability

Data is available after reasonable requests to the corresponding author.
